# When to Adjust for Multiple Testing: A Unifying Guiding Principle

**DOI:** 10.1002/bimj.70148

**Published:** 2026-07-02

**Authors:** Sabine Hoffmann, Simon Lemster, Gary Collins, Alexander Hapfelmeier, Georg Heinze, Andreas Mayr, Matthias Schmid, Juliane C. Wilcke, Anne‐Laure Boulesteix

**Affiliations:** ^1^ Department of Statistics LMU Munich Munich Germany; ^2^ Institute for Medical Information Processing, Biometry, and Epidemiology, LMU Medizin LMU Munich Munich Germany; ^3^ Department of Applied Health Sciences, School of Health Sciences, College of Medicine and Health University of Birmingham Birmingham UK; ^4^ Institute of AI and Informatics in Medicine, TUM School of Medicine and Health Technical University of Munich Munich Germany; ^5^ Institute of General Practice and Health Services Research, TUM School of Medicine and Health Technical University of Munich Munich Germany; ^6^ Institute of Clinical Biometrics, Center for Medical Data Science Medical University of Vienna Vienna Austria; ^7^ Institute for Medical Biometry and Statistics University of Marburg Marburg Germany; ^8^ Institute for Medical Biometry, Informatics, and Epidemiology University Hospital Bonn Bonn Germany; ^9^ Munich Center for Machine Learning (MCML) Munich Germany

**Keywords:** family‐wise error rate, good practice, guidance, hypothesis testing, multiplicity, reporting, Type I error

## Abstract

Most original articles published in the medical literature report the results of multiple statistical tests. In a few simple cases, there is agreement on whether to adjust for the number of performed tests. For many cases encountered in practice, however, this is less clear, and the recommendations in the literature are contradictory, along different dimensions, or otherwise confusing. This lack of clear guidance may impair the conduct and interpretation of analyses, and encourage questionable research practices, ultimately jeopardizing the credibility of medical research. In this article, we refine, illustrate, and discuss a unifying guiding principle to assist both statisticians and applied researchers in deciding whether to adjust for multiple testing and, if so, over which set of tests. The principle is that *multiple testing should be adjusted for if and only if authors, when reporting and interpreting their findings, put more emphasis on results of one or several of the tests because of their small p‐value(s)*. We relate this principle to previously proposed rules and show how it can guide and clarify the choice of adjustment strategies in three complex multiple testing settings.

## 1 Introduction

The problem of multiple testing has received considerable attention in the past three decades. The increasing use of complex adaptive designs for clinical trials as well as the advent of “extreme multiple testing” considering hundreds to hundreds of thousands of tests simultaneously (e.g., in molecular or imaging research) have fueled methodological developments related to multiple testing and inspired a variety of concepts and adjustment procedures. Researchers wondering which adjustment method they should use can rely on a wide body of solid statistical literature and ample guidance; accordingly, we do not address this topic here. In contrast, researchers wondering whether their study requires adjustment at all, and if yes, over which set of tests they should adjust, have limited literature to draw upon. In a few basic settings, there is agreement on whether to adjust for the number of performed tests. In many other situations, however, there is contradictory advice, if at all, that makes the decision dependent on several criteria, leading to confusion and uncertainty among researchers. Table 1 shows a selection of partly contradictory statements from the literature on the need to adjust for multiple testing.

**TABLE 1:** (When) should one adjust for multiple testing? Articles illustrating the diversity of opinions and arguments in the literature.

Reference	Field	Message in relation to the need to adjust
		**Do not adjust**
O'Keefe (2003)		Adjustment should be abandoned.
Perneger (1998)		“Bonferroni adjustments are, at best, unnecessary and, at worst, deleterious to sound statistical inference.” (p. 1236)
Rothman (1990)	Epidemiology	Not adjusting leads to fewer errors when the null hypothesis is false (i.e., fewer type II errors).
Feise (2002)	Clinical trials	One should select a primary endpoint or combine endpoints, or communicate the chances of making type I and type II errors to readers.
Althouse (2016)		One should report all tests that were done and “let readers use their own judgment about the relative weight of the conclusions.” (p. 1645)
		**Adjust in certain cases**
Cook and Farewell (1996)	Clinical trials	Adjustment is often not needed, especially if the trial is designed carefully and tests are interpreted marginally.
Proschan and Waclawiw (2000)	Clinical trials	The need to adjust depends on a combination of at least five factors: “the relatedness of the questions of interest, the number of comparisons, the degree of controversy, the possibility that a single entity may gain from multiplicity, and the nature of the study/alternative hypothesis.” (p. 534)
Bender and Lange (2001)	Epidemiology	Adjustment is needed “in confirmatory studies whenever results from multiple tests have to be combined in one final conclusion and decision.” (p. 343)
Streiner and Norman (2011)	Clinical trials	Adjustment is not needed when testing baseline variables, but needed in exploratory analyses, except if the exploratory character is clearly stated. For primary endpoints the answer depends on several factors.
Rutherford (2011)		Adjustment is needed for unplanned experiments.
Pocock (1997)	Clinical trials	There is no need to adjust in the analysis of secondary endpoints.
Parker and Weir (2022)	Clinical trials	There is no need to adjust in the analysis of secondary endpoints.
Schober and Vetter (2020)		Adjustment is not needed in conjunction testing and may not be needed in exploratory research.
Rubin (2021)		Adjustment is inappropriate in the case of conjunction and individual testing, but appropriate in the case of disjunction testing.
García‐Pérez (2023)	Psychology	Adjustment is only justifiable when testing a joint null hypothesis, for which a one‐shot statistical test does not exist.
Rubin (2024)	Psychology	Adjustment is needed only if the alternative hypothesis is a union hypothesis of the form 0“$H_{1,1}$ or $H_{1,2}$“.
		***Do adjust***
Goeman and Solari (2014)	Omics research	There is no reason not to adjust when testing omics markers.
Frane (2019)		Anti‐adjustment arguments from the literature are often based on misconceptions.

A frequently used criterion is the distinction between exploratory and confirmatory analyses. For example, Bender and Lange (2001) only recommend rigorous adjustment for multiple testing in confirmatory analyses, and consider it unnecessary in exploratory studies without prespecified hypotheses. However, others have argued that adjustment—if needed at all—is especially important for unplanned experiments (Althouse 2016; Rutherford 2011). Moreover, there are also confirmatory settings in which adjustment may not be necessary, such as in the analysis of secondary endpoints in randomized clinical trials (RCTs) (Parker and Weir 2022; Pocock 1977). Other authors also suggest to make the decision on whether to adjust for multiple testing dependent on the type of outcome variable considered (Streiner and Norman 2011), or on the way in which the multiple hypotheses are combined (disjunction vs. conjunction testing; Dmitrienko and D'Agostino 2013; Rubin 2021).

Even more intricate is the choice of the family of tests, that is, the set of hypotheses to adjust over—in case it has been decided to adjust. Althouse (2016) considers this choice as spurious, pointing to the absurdity of adjusting for all performed tests including those on baseline characteristics and to the intricate cases of studies consisting of clearly distinct parts or studies being complemented over time by additional substudies. Likewise, Proschan and Waclawiw (2000) and Hooper (2025) point to the situation where researchers perform additional tests using already published data and question whether they should adjust for the number of previously performed tests. The difficulty of defining what constitutes a family of tests or, in their terminology, an *experiment* (over which one has to adjust) is also noted by Bender and Lange (2001). The idea of tests belonging to one experiment is not easy to communicate, especially in observational research, making the identification of these families difficult in many situations. This problem appears to be underconsidered in the literature, and if considered, is often used as an argument against adjustment rather than as a basis for providing solutions.

If researchers adhere to the strict rule of always adjusting for multiple testing, they risk considerably reducing the statistical power of their studies. It can even be argued that recommendations to adjust may encourage the “salami‐slicing” of a research project into as many single‐hypothesis papers as possible (Althouse 2016; Perneger 1998). On the other hand, never adjusting for multiple testing increases the rate of false‐positive results, leading to overconfidence and non‐replicable findings.

In this paper, we illustrate and discuss a unifying guiding principle, which we recently sketched in an opinion piece published in a clinical journal (Boulesteix and Hoffmann 2024), to assist both statisticians and applied researchers in deciding whether to adjust for multiple testing and, if so, over which set of tests. We demonstrate its application in three complex multiple testing scenarios where no consensus currently exists within the community. The guiding principle helps with the choice of when to adjust, but this decision is essentially independent of the choice of how to adjust. As a consequence, it is applicable to all methods of multiple testing adjustment. For the sake of simplicity, we use the Bonferroni correction in the presented applications, although other methods may often be more suitable in practice.

## 2 Methods: The Decision to Adjust Should Be Related to the Reporting and Interpretation of Results

The decision to adjust for multiple testing should be inherently linked to how the significance of results affects the reporting and interpretation of the findings. The general principle is that *multiple testing should be adjusted for if and only if authors, when reporting and interpreting their findings, put more emphasis on the results of one or several of the tests because of their small p‐value(s)* (cf. Boulesteix and Hoffmann 2024).

This unifying principle builds on previous observations that the core problem lies not in the use of multiple tests per se, but in selective reporting. For example, Benjamini and Hochberg (2000) note that “conducting the analysis for many subgroups and highlighting or reaching decisions about the selected few that come out to be statistically significant raises a danger that the conclusions from the study will not be a result of a real phenomenon but merely reflect the selection of the extremes among the extensively tested noise.” In a similar vein, Cox (2006, p. 87) writes that “[m]any investigations set out to answer several questions via one set of data and difficulties arise not so much from dealing with several questions […] but rather from selecting one or a small number of questions on the basis of the apparent answer.” Building on these observations, the proposed guiding principle makes it clear that the question of whether one should adjust for multiple testing is only indirectly affected by the exploratory or confirmatory nature of an analysis, or even by the research question in itself. Instead, the decision depends on the reporting and the interpretation of the results of the multiple performed tests. If significance is the key factor determining which results are emphasized and which are relatively downplayed when reporting, discussing, and interpreting a study, it is necessary to adjust for multiple testing. If, regardless of their significance, the results of all tests are reported transparently with approximately equal emphasis and detail, it is not necessary to adjust and power can be saved.

This principle automatically addresses concerns by Bender and Lange (2001) and Althouse (2016) regarding the difficult choice of the family of tests over which one should adjust. Its corollary is indeed that a family of tests requiring adjustment consists of each set of tests for which selective reporting and interpretation occur and depend on significance. More formally, if the researcher ranks the *p*‐values of the m tests and puts more emphasis on the $m^{\prime }$ tests with the smallest *p*‐values (where $1\leq m^{\prime }<m$) and less emphasis on the $m-m^{\prime }$ other tests, the m tests form a set over which one should adjust. In contrast, if some of the m tests are reported independently of their ranking relative to the others, they should not be considered part of this set. This is typically the case for tests conducted for purposes such as controls, plausibility checks, or complementary analyses, which generally do not require adjustment. Tests that are not included in a given set requiring adjustment—because they are reported independently of their significance relative to the other set members—may still belong to a different set; see Showcase 2 (Section 3.3) for an example involving two sets corresponding to different types of molecular variables.

In some cases, the research question naturally leads to families of tests. Multiple primary endpoints in a clinical trial are an example of a natural family. When only one among these endpoints shows a significant treatment effect, it will typically be reported with more emphasis in a research paper. The results of tests for safety endpoints, on the other hand, have no influence on the reporting of primary endpoints and are usually reported with equal detail and emphasis, no matter whether the respective null hypothesis was rejected. According to the guiding principle, it is natural to consider the tests performed on the primary endpoints as a set of tests over which one should adjust, whereas the tests on safety endpoints do not form a set over which adjustment is necessary. In other cases, however, the decision on the set of tests over which one should adjust is implicitly made through the reporting and the interpretation of results. In such a delicate situation, we will see through examples in Section 3 that the guiding principle may be particularly helpful to decide over which set of hypotheses to adjust. We will also illustrate in these showcases that unadjusted results will be misleading unless researchers put approximately equal emphasis on all tests performed, both in the reporting and in the interpreting of their results.

At first glance, the guiding principle we propose appears to be quite similar to the recommendation by Rubin (2021) to not adjust in the case of “individual testing, in which *each* individual result must be significant in order to reject each associated individual null hypothesis” (p. 10969), and that by Bender and Lange (2001) to adjust for multiple testing “whenever results from multiple tests have to be combined in one final conclusion and decision” (p. 343). In many cases, the set of tests from which a single conclusion will be drawn is the same as the one among which selective reporting occurs, and our guiding principle coincides with the recommendation by Bender and Lange for confirmatory research. However, we argue that the recommendations by Rubin as well as Bender and Lange can be gamed by claiming that tests are performed to test individual hypotheses that will not be combined in one final conclusion.

In a hypothetical example suggested by one of the reviewers, a pharmaceutical company could, for instance, assess three doses of a drug in a clinical trial and then argue that they are in fact testing the three individual null hypotheses “There is no treatment effect at dose 1,” “There is no treatment effect at dose 2,” and “There is no treatment effect at dose 3.” If they find a significant result for dose 3, but not for doses 1 and 2, they could then claim that, in line with Bender and Lange (2001), they make the following three individual conclusions and subsequent decisions: “Dose 1 should not be marketed,” “Dose 2 should not be marketed,” and “Dose 3 should be marketed.” Similarly, according to Rubin (2021), they would not need to adjust for multiple testing because they are testing individual null hypotheses (see Lakens, 2022, for a very similar line of argumentation in Chapter 2). According to the guiding principle we propose, however, they would need to adjust because they put more emphasis on the results for dose 3 in the interpretation of their results by proposing dose 3 for marketing. More precisely, the pharmaceutical company could either choose a neutral reporting strategy with equal emphasis on all performed tests. A reporting strategy that would be as neutral as possible is “the unadjusted *p*‐values of the performed tests were x, y, and z.” In this case, however, it would be incoherent to conclude that dose 3 should be marketed. If, on the other hand, they choose to interpret their findings as showing that dose 3 worked (in order to be able to conclude that dose 3 should be marketed), they clearly put more emphasis on the results of the test for dose 3 in the interpretation of their results because of its significance, by suggesting a practical change for dose 3 but nothing particular for doses 1 and 2. According to our guiding principle, they would therefore need to adjust their results for multiple testing in this second case.

In this context, it is important to emphasize that the guiding principle we propose explicitly relates the decision to adjust for multiple testing to both reporting and interpretation, where interpretation may include potential recommendations and consequences derived from the test results (as in this hypothetical example). In particular, if the text describing the results explicitly mentions the results of all tests (in our example “dose 1 was not significant, dose 2 was not significant, and dose 3 was significant”), this arguably also qualifies as putting equal emphasis on all results. However, there is a risk that the authors (and readers) will put more emphasis on the significant results in their interpretation, and when this happens adjustment is necessary. To decide whether the interpretation of the reported results puts equal emphasis on all tests, one can check whether it is logically coherent with the more neutral reporting strategy “the unadjusted *p*‐values of the performed tests were x, y, and z.” Finally, note that the family‐wise error rate (and the false discovery rate) will of course be higher than the alpha level whenever multiple tests are performed. However, when using a neutral reporting‐and‐interpretation strategy, this is actually irrelevant, because it is not possible to reject individual null hypotheses. As soon as researchers reject an individual null hypothesis, they put more emphasis on this test result because of its significance and therefore would need to adjust according to the guiding principle we propose.

## 3 Results: Using the Guiding Principle in Practice

The guiding principle first sketched by Boulesteix and Hoffmann (2024) and detailed above can be useful in various phases of research, particularly in the design, analysis, reporting, and interpretation of studies. In this section, we first discuss how this principle generalizes well‐known rules in established cases and then show how it can provide answers in more complex situations.

### 3.1 Established Cases

The guiding principle is *unifying* in the sense that it is not a complex set of rules that depend on the type of study, and that it is in concordance with the generally recommended line of conduct in several simple cases of multiple testing for which such a well‐established agreement exists in the literature (see Table 2). While the general recommendations along with selected references are given in the table only, we briefly describe the five settings below and explain in each case how the guiding principle leads to the decision to adjust or not.

**TABLE 2:** Using the guiding principle in five simple cases: Column 2 summarizes the common recommendations from the literature, while Column 4 contains the recommendation derived from the guiding principle.

Setting	Common recommendation	References	Guiding principle
1. Multiple primary endpoints in RCT	If no other option, such as composite endpoints, is possible and the RCT is declared successful as soon as at least one of the tests is significant → **Adjust**	Dmitrienko and D'Agostino (2013) FDA (2022)	More emphasis on significant result → **Adjust**
2. Interim analyses in RCT	→ **Adjust** using a dedicated procedure (e.g., Pocock)	Pocock (1977)	Only first significant result counts, i.e. more emphasis on first significant result → **Adjust**
3. Secondary endpoints in RCT	→ **Do not adjust**	Pocock (1977) Parker and Weir (2022)	Results reported on equal footing → **Do not adjust**
4. Large number of endpoints (e.g., in omics experiments)	When testing, say, 20,000 biomarkers for association with an outcome of interest, 1000 of the corresponding null hypotheses are expected to be rejected at α=0.05, even if all are true → **Adjust**	Dudoit et al. (2003) Goeman and Solari (2014)	Only results with the smallest p‐values reported → **Adjust**
5. Multiplicity of analysis strategies	Try to report, reduce, accept, or integrate uncertainty; otherwise → **Adjust**	Hoffmann et al. (2021) Mandl et al. (2024)	Selection of strategies with most significant results → **Adjust**

Let us first consider an RCT with multiple primary endpoints (Setting 1) that is declared and reported as successful as soon as at least one of the tests is significant, even if no success was observed for the other endpoint(s). This means that there is more emphasis on the endpoint for which the null was rejected—precisely because of the rejection. According to the principle, one should adjust for multiple testing.

The same can be said of a clinical trial with interim analyses (Setting 2), which is stopped and declared successful as soon as the null‐hypothesis is rejected in an interim analysis. Again, more emphasis is placed on the test with a significant result than on the previously performed tests with a nonsignificant result, in the sense that the trial is considered successful even if the first tests did not reject the null hypothesis. According to the guiding principle, one should adjust for multiple testing, which can be done in practice using group‐sequential designs (O'Brien and Fleming 1979; Pocock 1977).

In contrast, the results of the tests for the secondary endpoints of an RCT (Setting 3) are typically reported on equal footing no matter whether the respective null hypothesis was rejected or not. According to the guiding principle, one should not adjust for multiple testing.

In the case of high‐dimensional omics data (Setting 4), authors typically report and discuss only a small proportion of the many tests performed—namely, those yielding the smallest *p*‐values. They may, for example, restrict their reporting to tests with adjusted *p*‐values below the significance threshold or to a fixed number of top‐ranking tests, while omitting others, including some that also reach significance. This fixed number may, for instance, depend on the resources available for follow‐up wet‐lab experiments. Clearly, these tests are reported precisely because of the *p*‐values they yield. According to the principle, one should therefore adjust for multiple testing.

When authors perform many different analysis strategies and selectively report the results of those yielding the most significant results in their paper (Setting 5), they put more emphasis on the most significant results when writing their manuscript. According to the principle, adjustment is necessary. This is consistent with the literature, which emphasizes the need to account for the multiplicity of analyses in some way. In practice, this may be achieved through formal adjustment for multiple testing in a strict sense (Mandl et al. 2024), but not necessarily so. See Hoffmann et al. (2021) for an overview of potential solutions, including additionally reporting alternative results as well as aggregating the multiple results in some way.

The next three subsections aim to demonstrate, using three illustrative examples, that the guiding principle is not only in agreement with various general recommendations, but also yields new insights in the form of clearer answers for more complex situations, which go beyond the simple cases discussed in the literature.

### 3.2 Showcase 1: Power Calculation for an Animal Experiment

In the design of an exploratory animal experiment, a large number of comparisons and tests may be planned. For example, a group of researchers may plan to compare the effect of two treatments (A and B) against placebo on two outcomes at four time points and three dosing levels in two groups of mice defined by disease severity. In this situation, researchers might decide that they need to adjust for the total number of 2×2×4×3×2=96 tests (resulting in a loss of power or an unethically large number of animals) or that they do not need to adjust at all (resulting in Type I error inflation). The guiding principle helps to avoid these two suboptimal strategies by identifying the set of tests over which adjustment should be performed when planning a study.

The researchers may, for instance, know from disease pathology and previous experiments that higher dosing will always lead to a larger effect and that the treatment is much more likely to work in the group of mice with low disease severity than in the group with high disease severity. Additionally, treatment A may be an established treatment that has been shown to be effective to treat the disease in many previous experiments and, in this case, it may have been included in the experiment as a positive control for which the effects are known. As a consequence, the significance of any tests involving treatment A and the different dosing levels will not influence the reporting of results—in the sense that these tests will not receive more attention because of significant results: For treatment A, it is not interesting to put more focus on significant results because the effectiveness of treatment A has already been established. For the dosing level, any patterns where there are significant results for low dosing levels but not for high dosing levels will be considered as biologically not plausible. Similarly, there will not be more focus on the results for the group of mice with high disease severity if the tests for the group with low disease severity are not significant, again because such a finding would be biologically implausible. The researchers would rather conclude that their experiment has failed in some way if the treatment shows significant improvements in the severe group and at low dosing levels, but not in the mild one and at high dosing levels. On the other hand, if the tests for outcome 1 are significant, but not for outcome 2, the researchers will probably mention the former result in the abstract and even in the title of the paper, whereas they would not dwell too much on outcome 2 in the interpretation of their results. Similarly, they might conclude that the treatment has shown overall effectiveness even if they only observed a significant result for one or two time points.

In this scenario, the guiding principle helps to reduce the number of tests for which to adjust from 96 to eight, as the four time points and two outcomes are the only tests for which authors put more weight on significant results because of their significance when reporting, discussing and interpreting their findings. If one performs power calculations assuming—for simplicity—that the Bonferroni procedure will be used for adjustment (which would be very conservative in this situation, given that the two outcomes and the tests at different time points are probably correlated), one would set the α level to 0.05/8 for those eight tests of main interest. Depending on the relevance of the risk of making Type II errors, one might additionally want to check for some of the other tests (or all) if they are sufficiently powered at an α level of 0.05.

A variant of this example would be a 2×2 factorial design involving the two treatments A and B (and their respective placebos), with A being an established treatment serving as positive control as discussed above. In this case, the conclusion that treatment A works would not be worth emphasizing, and the corresponding test would not be part of the family one has to adjust over. However, if “treatment B works” (main effect of B) and “the effect of treatment B is stronger when the patient also receives A” (interaction effect) are both conclusions that may be worth focusing on, adjustment should be performed. Overall, this example illustrates that the question of how the results will be reported and interpreted allows to clarify the adjustment strategy for multiple testing in the design phase of an experiment.

### 3.3 Showcase 2: Two Groups of High‐Dimensional Variables

**FIGURE 1:**
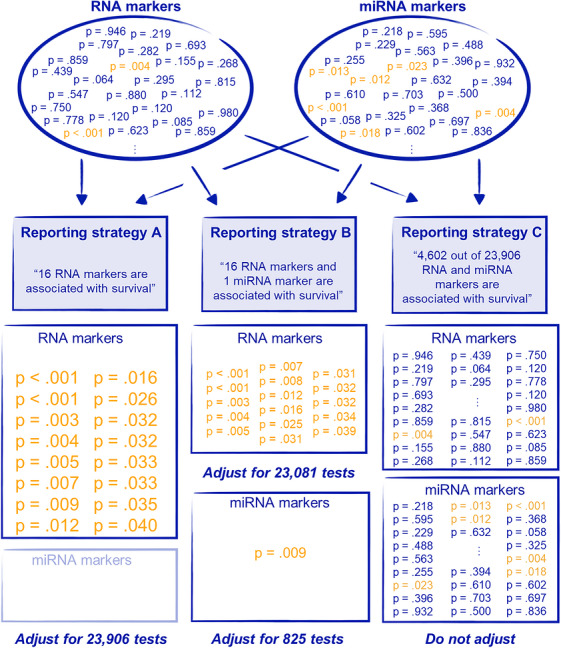
Application of the guiding principle to different possible reporting strategies for the association between two types of molecular markers and overall survival. Researchers may focus their reporting on the marker type yielding overall stronger associations with the outcome and adjust over all markers (strategy A), report the significant markers for both types and therefore adjust separately (strategy B), or report all markers on equal footing and not adjust at all (strategy C).

In the analysis of the association between molecular markers measured using high‐throughput techniques and clinical outcomes, an initial screening is commonly performed using univariate tests to decide which markers require further investigation. The following example uses data from 120 patients with urothelial bladder carcinoma, publicly available from The Cancer Genome Atlas (TCGA, Weinstein et al. 2013) and preprocessed as in Herrmann et al. (2021). The considered outcome is overall survival, and the candidate markers are 23,081 RNA markers and 825 miRNA markers. We used univariate Cox regression models to assess the association between survival and each of the markers and—for simplicity—the Bonferroni procedure for adjustment (note that, in practice, it may also make sense to use a procedure controlling the false discovery rate, see Benjamini and Hochberg 1995). The different possible reporting strategies and adjustment approaches derived from the guiding principle are schematically represented in Figure 1. If the authors want to focus on the marker type RNA when reporting the study because it shows, roughly speaking, “a stronger association with the outcome,” while giving less attention to the other marker type, according to the guiding principle, adjustment should be performed over the 23,081+825 markers simultaneously (strategy A). In contrast, if they want to report the results for the RNA and the miRNA markers on equal footing, independently of the test results, adjustment can be performed within each type of marker, and the null can then be rejected for one of the miRNA markers (strategy B). No adjustment at all is necessary according to the guiding principle if they report all 23,081 and 825 test results on equal footing (strategy C), but this would result in a tedious and confusing paper.

### 3.4 Showcase 3: Several Novel Potential Risk Factors

In a cohort study, one may be interested in the research question of whether there is an association between all‐cause mortality and the blood levels of various vitamins (e.g., vitamins A, B9, B12, C, D, E, K). When addressing this research question, it is important to adjust for several confounding factors, such as age, sex, physical activity, smoking status, alcohol consumption, body mass index, and other dietary covariates. Irrespective of the statistical approach that is chosen to adjust for confounding in this situation (one may choose to include all variables in a logistic or Cox regression model or use a more sophisticated approach to avoid the so‐called Table 2 fallacy; Westreich and Greenland 2013), we can obtain an estimate of the association with all‐cause mortality along with a *p*‐value for every vitamin and every adjustment variable.

In this multiple testing situation, a researcher may typically choose between adjusting for the total number of tests, or not adjusting at all. According to the guiding principle, however, one would adjust for the set of tests on which the authors put more emphasis when reporting, discussing, or interpreting their findings if these tests yield significant results. In this example, this set would only include the vitamins, while the other covariates, for which the association with the outcome of interest is already well established in the literature, have no impact on the reporting and interpretation of results. A significant association between age and overall mortality would not translate into the title “Age is related to overall mortality” or the mentioning of this significant association in the abstract. The guiding principle thereby clearly indicates that it is not necessary to adjust for potential tests that may be performed for adjustment variables (e.g., to verify that the associations of these confounders with the outcome is as expected based on clinical knowledge).

When it comes to the group of vitamins, two reporting strategies are possible. Authors can put more emphasis on the variables that turned out to be significant, for instance, by writing in the title “Vitamin D and vitamin B12 are independent risk factors of all‐cause mortality” in the case where only these two vitamins turned out to be significant. Alternatively, authors can choose a more neutral and less catchy interpretation, avoiding any *spin*, by reporting that “Two out of seven vitamins are independent risk factors for all‐cause mortality.” In the first case, the guiding principle indicates that it is necessary to adjust for the fact that seven tests were performed, whereas this is not necessary in the second case, because the authors put equal emphasis on all performed tests, irrespective of their significance. While the second interpretation provides less information on the exact vitamins that are associated with all‐cause mortality, it does allow for the evaluation of the overall signal‐to‐noise ratio and importance of the group of vitamins for the outcome of interest.

## 4 Discussion

In this paper, we illustrated and discussed the general guiding principle sketched by Boulesteix and Hoffmann (2024), which builds on previous observations that the core of the problem does not lie in multiple testing per se, but in selective reporting and interpretation. This principle provides a framework to guide researchers on when to adjust and, if so, over which set of tests, beyond trivial situations. It does not make this decision easy or self‐evident, but certainly less difficult. It is unifying in the sense that it subsumes several widely accepted recommendations as special cases. We demonstrated its application in three case studies. To avoid complicating the message, we refrained from addressing the question of how to adjust and focused primarily on the well‐known Bonferroni procedure for controlling the family‐wise error rate. However, we acknowledge that more powerful alternative procedures (in particular, those taking the dependence pattern of hypotheses into account) or error concepts (such as the false discovery rate, Benjamini and Hochberg 1995) may be preferable in many settings.

The guiding principle also makes clear, beyond the context of adjustment for multiple testing in a narrow sense, that publication bias should be adjusted for when conducting meta‐analyses. Using our terminology, we can say that the scientific literature as a whole tends to place greater emphasis on significant than on nonsignificant results through the action of journal editors and reviewers (who are more likely to reject studies with negative results) or of authors themselves (through self‐censorship in case of disappointing results). One thus has to adjust for publication bias when estimating an effect in meta‐analysis, and this can be seen as an adjustment for multiplicity in a broad sense, even if the methods to be used to correct for publication bias are not the same as those used for multiple testing correction in the classical sense.

However, the guiding principle also has a number of limitations. First, the phrase “placing more emphasis” is open to subjective interpretation, especially since overall emphasis is shaped by the wording, tables, and graphics throughout the article. It is obvious that a test that is specifically mentioned in the title or the abstract of a paper receives more emphasis than other tests. However, it may be more difficult to judge whether the authors give more attention to the interpretation of different tests in the results and discussion section or through the use of figures. Placing more emphasis is not a binary feature, and in practice one cannot expect all tests to receive *exactly* the same level of emphasis. The guiding principle concerns the overall message rather than a literal count of words; it should therefore not be interpreted in a narrow or overly strict sense.

Second, the principle does not protect against self‐deception. Except in the case of pre‐registered studies, it will often be possible for researchers to convince themselves and others that they intended to focus on a specific result in the first place and that they did not change their reporting‐and‐interpretation strategy after seeing the results. Thirdly, the guiding principle is not of any help if statistical testing is performed in a spurious way, for example, in the hope of confirming the null‐hypothesis. This is typically the case when a new treatment is compared to a standard treatment with respect to safety endpoints. In this situation, however, the guiding principle could in principle be used in the context of equivalence testing.

Finally, even if researchers report and interpret all tests with equal emphasis irrespective of their significance in a paper, readers may selectively report and interpret findings from the paper, often putting more emphasis on significant results. This risk can be viewed as a limitation of the guiding principle. To mitigate it, it may make sense to report not only the unadjusted (raw) *p*‐values in the original paper, but also adjusted *p*‐values for the family (or families) of tests most likely to be of interest, and to advise readers who choose to selectively consider, re‐report, or interpret findings to rely on correspondingly adjusted *p*‐values. In this context, we underscore that no criterion or reporting strategy can substitute for the individual responsibility of the reader, nor can it render statistical literacy dispensable.

Going one step further, the basic idea of the guiding principle may be useful not only to authors who have to decide whether to adjust, but also to readers who select results based on their statistical significance from a number of studies or papers for further study. Such an extension of the guiding principle may be further elaborated in future work. It would address cases such as authors trying to game the guiding principle by splitting their study into several papers and readers selecting one of them for further investigation of its results.

While the guiding principle is based on how the results are intended to be reported, an issue that we have not addressed is the reporting of the applied correction itself. Reporting guidelines such as STROBE for observational studies (Vandenbrouckel et al. 2007) contain no recommendations on what to report, whilst SPIRIT (Chan et al. 2025) and CONSORT for randomized trials (Hopewell et al. 2025) are relatively vague and make no firm recommendations on what to report when it comes to multiple testing. For example, at the trial design phase, the SPIRIT‐2025 reporting guidelines for protocols of RCTs (item 27a) list “Methods to account for multiplicity, if applicable” as a key element to address (Hróbjartsson et al. 2025). Similarly, at the trial results stage, CONSORT‐2025 (Hopewell et al. 2025) includes the following recommendations: “Any methods used to mitigate or account for multiplicity should be described. If no methods have been used to account for multiplicity (e.g., not applicable, or not considered), then this should also be reported, particularly when a large number of analyses has been carried out.” Furthermore, reporting guidelines, such as SPIRIT and CONSORT, only mention multiplicity in the accompanying Explanation and Elaboration documents, which may go unread, and this may need revisiting in future updates to these reporting guidelines. To facilitate transparency and trust in research findings, consensus‐driven recommendations on key details to report on adjustments for multiple testing are urgently needed, given the breadth of available adjustment procedures. These may concern, for example, which correction or adjustment procedure was used (and why), whether hierarchical testing was applied, which outcomes, subgroups, and analyses were included in the adjustment, and the total number of tests performed.

Finally, it is important to emphasize that the guiding principle does not resolve the many misunderstandings (Goodman 2008; Greenland et al. 2016) and problems that may arise from the use of statistical significance in the medical literature (Amrhein et al. 2019l Wasserstein et al. 2019). What it can do is to help clarify when to adjust for multiple testing and to improve researchers' use, interpretation, and reporting of significance tests when they choose to rely on testing. By promoting this guiding principle, we do not mean to defend the use of significance testing itself. Importantly, the core idea of the guiding principle—that the need for adjustment is determined by the reporting strategy—extends beyond hypothesis testing to other contexts. For instance, it may help researchers to decide whether confidence intervals or Bayes factors—commonly advocated as alternatives or complements to null hypothesis significance testing—require multiplicity adjustment if the definition of the principle is rephrased as putting more emphasis on results of one or several quantities because of their extreme values.

## Funding

This study was partly funded by the German Research Foundation (DFG), grant BO3139/7‐2 to ALB.

## Conflicts of Interest

The authors declare no conflicts of interest.

## Open Research Badges

This article has earned an Open Data badge for making publicly available the digitally‐shareable data necessary to reproduce the reported results. The data is available in the Supporting Information section.

This article has earned an open data badge “**Reproducible Research**” for making publicly available the code necessary to reproduce the reported results. “The results reported in this article could fully be reproduced.”

## Supporting information


**Supporting File:** bimj70148‐sup‐0001‐Data.zip.

## Data Availability

The code implementing the analysis for Showcase 2 is available from the supplement.
